# A Visual Servoing-Based Method for ProCam Systems Calibration

**DOI:** 10.3390/s131013318

**Published:** 2013-10-01

**Authors:** Francois Berry, Omar Ait Aider, Jeremie Mosnier

**Affiliations:** DREAM, Insitut Pascal, 24 avenue des Landais, Aubiere 63170, France; E-Mails: omar.ait-aider@univ-bpclermont.fr (O.A.A.); jeremie.mosnier@gmail.com (J.M.)

**Keywords:** projector calibration, structured light system, OpenGL modeling, visual servoing

## Abstract

Projector-camera systems are currently used in a wide field of applications, such as 3D reconstruction and augmented reality, and can provide accurate measurements, depending on the configuration and calibration. Frequently, the calibration task is divided into two steps: camera calibration followed by projector calibration. The latter still poses certain problems that are not easy to solve, such as the difficulty in obtaining a set of 2D–3D points to compute the projection matrix between the projector and the world. Existing methods are either not sufficiently accurate or not flexible. We propose an easy and automatic method to calibrate such systems that consists in projecting a calibration pattern and superimposing it automatically on a known printed pattern. The projected pattern is provided by a virtual camera observing a virtual pattern in an OpenGL model. The projector displays what the virtual camera visualizes. Thus, the projected pattern can be controlled and superimposed on the printed one with the aid of visual servoing. Our experimental results compare favorably with those of other methods considering both usability and accuracy.

## 1. Introduction

3D reconstruction has been in use for more than 30 years and is still an active research topic in the computer vision community. One of the reasons for the popularity of such systems is the large application field they cover, such as manufacturing, security systems, medical systems and games. Surface reconstruction can be divided into two different categories. The best known example of the first class, in which we find passive scanners, is stereovision, which consists of two or more cameras viewing a scene from different angles. A set of frames is acquired, and 3D information can be extracted by triangulation after resolution of the 2D–3D correspondence problem. The major drawback of this technique lies in establishing correspondences, in particular, with textureless scenes.

To overcome such difficulties, new techniques called active scanners were developed 20 years ago. In these techniques, an emitter source is used, such as light, X-ray or ultra-sound. One of the methods of this class is called time-of-flight (TOF). It uses a laser to emit a pulse of light, and the amount of time before the reflected light is seen by a detector is timed. The biggest drawback is the time processing. Another method for active scanners uses structured light. This technique consists in augmenting the 3D scene by the projection of a coded pattern. The pattern is detected in the images, and the 3D reconstruction can be achieved by triangulation. The coding of the pattern is crucial in retrieving 3D information with robustness. Salvi *et al.* [1] classified these coding strategies into three groups of patterns: time-multiplexing, spatial neighborhood and direct coding. Time-multiplexing and direct codification are preferentially limited to static scenes, while the spatial neighborhood is focused on moving scenes. Readers could also refer to Zhang and Li [2], who give an up-to-date review of structured light techniques.

As reported in many articles, the accuracy of such systems greatly depends on the calibration task. Calibration consists in computing intrinsic parameters of both the camera and the projector and the 3D transformation between them. The biggest challenge in camera–projector calibration is the expression of 2D–3D correspondences for the projector. At present, several different methods exist to accomplish this task, and they can be separated into two groups. There are methods that first calibrate the camera, then the projector and deduce the rigid motion matrix between the two devices. One classical method is to use a printed checkerboard pattern as a projection plane [3–6]. The attached checkerboard is used to compute the orientation and the position of the plane in the camera frame, after which the ray–plane intersection is computed to recover the 3D position of each projected corner. With these points, the intrinsic parameters of the projector and the extrinsic parameters of the system can be deduced using classical monocular pose computation methods, such as in [7]. The main drawback of this method is that the error of the projector calibration depends directly on the accuracy of the camera calibration. Interested readers can also find a calibration toolbox of this method [5,8]. Another possibility, proposed by Shen and Menq [9], is to mechanically control the projection plane (no attached checkerboard is needed) to simplify the computation of 3D projected points.

There are other methods that integrate both intrinsic and extrinsic calibrations. Zhang and Huang [10] proposed a new approach for structured light system calibration by allowing the projector to acquire images as a camera would do and, thus, calibrate the projector independently of the camera. The key idea is to rely on a camera especially dedicated to acquiring “projector images”. By establishing correspondences between the camera and projector pixel (by a phase-shifting method), they transform the camera frame into a projector frame. Chen *et al.* [11] used a translating artifact of calibration to limit the number of views needed and a gray-coded and phase shifting pattern to solve the matching problem. Drouin *et al.* [12] proposed a large calibration pattern to take only one view using four cameras. They also devised an energy formulation to simplify the process of finding correspondences. Gao *et al.* [13] used a calibration board—a paper sheet (attached to a surface plane) with circular control points. A reference pattern with horizontal red stripes and one green stripe is projected onto the surface plane by a liquid crystal display (LCD) projector. 2D–3D point pairs for the projector and camera are obtained from epipolar geometry and the cross product. For most of the above methods, there are numerous constraints in processing calibration. For instance, with techniques that use a coded pattern or attached checkerboard, the calibration plane (*i.e.*, the surface of projection) must be physically unchanged: the authors assume that the scene is completely static during acquisition. Other methods use special calibration devices, such as a mechanically controlled plane or 3D artifacts.

Some authors have proposed alternative solutions to overcome these constraints. Recently, Drareni *et al.* [14] proposed a new calibration method for projectors with an uncalibrated camera. They got around the problem of determining the world–camera homography by looking for one that minimizes the reprojection error. They demonstrate that good accuracy can be achieved with fast time processing without the need for any additional devices. Audet and Okutomi [15] proposed a user-friendly application for calibration. Their method does not need a static scene, since the board can be manually placed in front of the system. They use printed fiducial markers (initially developed for augmented reality) that are completed by similar projected patterns. To align projected markers with printed ones, they compute a “prewarp” of the projected pattern consisting of an estimated homography.

**Figure 1.**
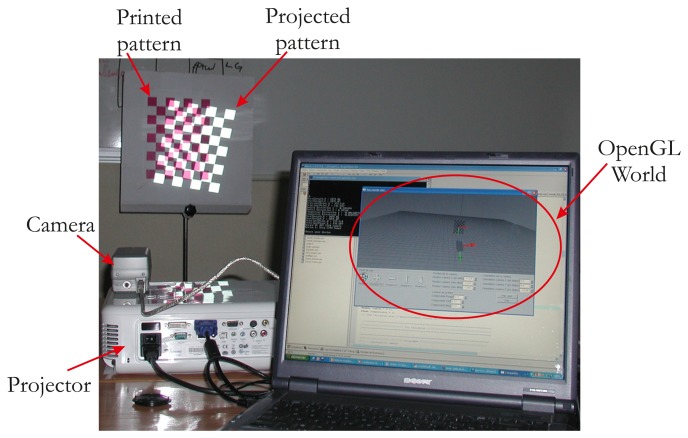
Setup of the system.

In this paper, we describe an automatic calibration technique that is easy to implement and does not require any additional devices and a static scene. The first study dedicated only to intrinsic parameters estimation of the projector has been published in [16]. We propose here a full and extended version of the method, which provides all calibration parameters (intrinsic and extrinsic parameters of the projector). We show that the calibration of the projector can be simply achieved starting from a calibrated camera [17] and a known pattern. Our idea is based on the assumption that the projector can be considered an inverted camera. The major innovation is the use of a visual servoing loop to make an automatic correspondence 2D/3D in the field of ProCam (Projector-Camera) calibration. This impacts on the usability of our method. A classical pinhole model can therefore be used, and calibration can be performed as it would be with a camera. Hence, using OpenGL, we model the projector as a virtual camera that observes a checkerboard pattern (Figure 1).

**Figure 2.**
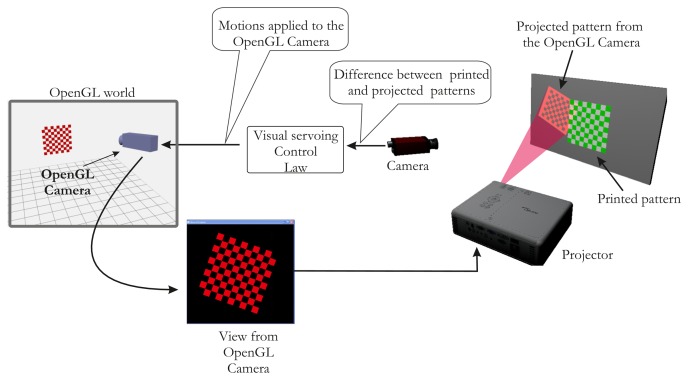
Overview of the setup: The virtual camera looks at a virtual printed pattern displayed by the projector.

Here, the projector is viewing like a moving camera, which allows us to make the pattern of translation and rotation. The projector displays the same intrinsic parameters what the virtual camera sees. Thus, our method consists in projecting a calibration pattern and superimposing it on a known printed pattern with the aid of visual servoing (by moving the virtual camera, Figure 2). The same 3D points (*i.e.*, the world points of the printed pattern) can be used for the camera and the projector, which considerably simplifies the process of projector calibration. In addition, the visual servoing task is able to minimize reprojection error on each point. A literature review showed that the accuracy of our method compares favorably with that of other techniques.

The paper is structured as follows. Section 2 describes the system model and the calibration method, Section 3 discusses visual servoing control loop, and in Section 4, we compare our method with existing techniques.

## 2. Projector and Camera Models

In our calibration method, system calibration is separated into two sequenced procedures: camera calibration and projector calibration. Camera calibration, using Zhang's method [10], is based on the reference data composed of the 3D reference points and their 2D camera correspondences. The same approach is used for the projector, but the difficulty lies in recovering the 3D reference points and their 2D projector correspondences. This challenge is the central feature of our work.

Calibration of a perspective projection system (such as a camera or projector) consists of estimating the intrinsic and extrinsic parameters and in defining the rigid motion between the two devices.

### 2.1. Perspective Projection Model

**Figure 3.**
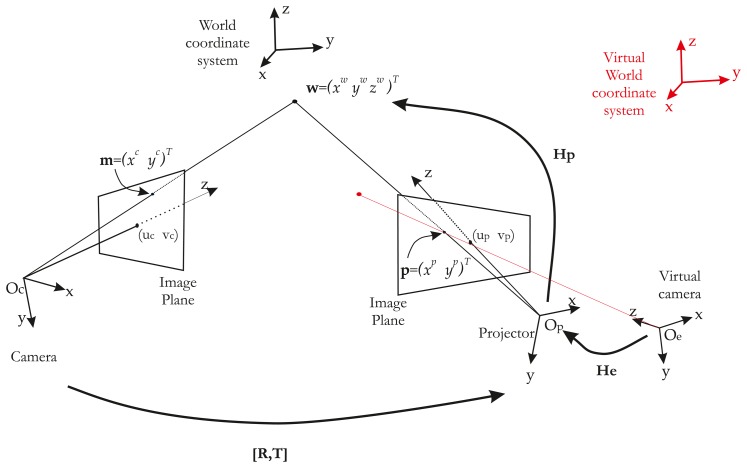
Projector-camera system modeling. A classical pinhole model is used for both devices. The two systems are related by the rigid motion matrix, [**R,T**].

Let us consider a point, **w**, with the coordinates, (*x^w^y^w^z^w^*)*^T^*, expressed by **w˜** in homogeneous coordinates (Figure 3). Let **m˜** = (*x^c^y^c^*1)*^T^* be its projection on the image plane. The relationship between **m** and **w** is given by the projection matrix, **H_c_**:

(1) $$\tilde{m}∼{\text{H}}_{\text{c}}\tilde{w}∼{\text{A}}_{\text{c}}[{\text{R}}_{\text{c}}|T_{c}]\tilde{w}$$

where ∼ means equality up to scale, **H_c_** is a 3×4 matrix and **R_c_** and **T_c_** describe, respectively, the 3×3 rotation matrix and the 3×1 translation vector. The location of O_c_ in the world coordinate system is given by translation **T_c_**:

(2) $${\text{T}}_{\text{c}}=\left[\begin{array}{c} t_{x}\\ t_{y}\\ t_{z} \end{array}\right]$$

The rotation between the camera and the world frames is defined by matrix **R_c_**

(3) $$R_{c}=\left[\begin{array}{ccc} R_{11} & R_{12} & R_{13}\\ R_{21} & R_{22} & R_{23}\\ R_{31} & R_{32} & R_{33} \end{array}\right]=\left[\begin{array}{c} r_{1}\\ r_{2}\\ r_{3} \end{array}\right]$$

**A_c_** represents the intrinsic parameters of the camera, such as:

(4) $$A_{c}=\left[\begin{array}{ccc} \alpha_{u_{c}} & \gamma_{c} & u_{0c}\\ 0 & \alpha_{v_{c}} & v_{0_{c}}\\ 0 & 0 & 1 \end{array}\right]$$

with *α_uc_* = *f .k_u_*, *α_vc_* = *f .k_v_* and *γ*_c_ = *f .k_u_*. cos *θ*.

The intrinsic parameters are:

- Focal lens *f*, which gives the distance between the image plane and the optical center, *O_c_*.
- *k_u_* and *k_v_*, giving the number of pixels per mm along *u* and *v* directions.
- (*u*_0_*_c_*,*v*_0_*_c_*) principal points in pixels.
- *θ*, which describes the non-orthogonality of rows and columns on the image sensor. This factor is usually negligible and set to zero.

The extrinsic parameters are defined by [**R_c_** | **T_c_**].

The projector is modeled like an inverted camera. Thus, its intrinsic and extrinsic parameters are similar to those of the camera. Consequently, point **p˜** = (*x^p^y^p^*1)*^T^* (on the image plane of the projector) is projected onto the scene in **w** = (*x^w^y^w^z^w^*)*^T^*. Thus, the relationship between **p** and **H_p_** is given by:

(5) $$\tilde{p}∼H_{p}\tilde{w}∼A_{p}[R_{p}|T_{p}]\tilde{w}$$

### 2.2. Rigid Motion between the Projector and Camera

The 3D transformation (expressed in the camera frame) between the two devices is given by:

(6) $$R=R_{p}{\left(R_{c}\right)}^{T}$$

(7) $$T=T_{p}+RT_{c}$$

Thus, the estimation of **R** and **T** is based on the extrinsic parameters of the projector and camera. Recovering these parameters consists in calibrating the camera–projector pair, which is similar to a stereo-rig system. In stereovision systems comprising two cameras, the calibration procedure can be described as follows:

- **Calibrating each camera**: Generally, a checkerboard printed pattern is attached to a plane surface, and a camera acquires images from different orientations. First, the printed pattern is used to identify 3D points with their projections in the image plane. By means of a set of acquired images, 2D–3D correspondences are extracted and used to compute the projection matrix **H_c_** using singular value decomposition (SVD). Second, the intrinsic and extrinsic parameters of the camera are extracted from **H_c_** [17].
- **Establishing 2D–3D correspondences for the two devices**: With the two calibrated cameras, another set of images is acquired with a printed checkerboard pattern attached to a plane. A set of image pairs are taken from different poses. The images are “corrected” using the distortion parameters. The 2D–3D correspondences are then extracted.
- **Computing the rigid motion**: Since the intrinsic and extrinsic parameters of both devices are known and with a set of 2D-2D correspondences, the rigid motion can be computed using the previous relationship (Equation (6)).

**Figure 4.**
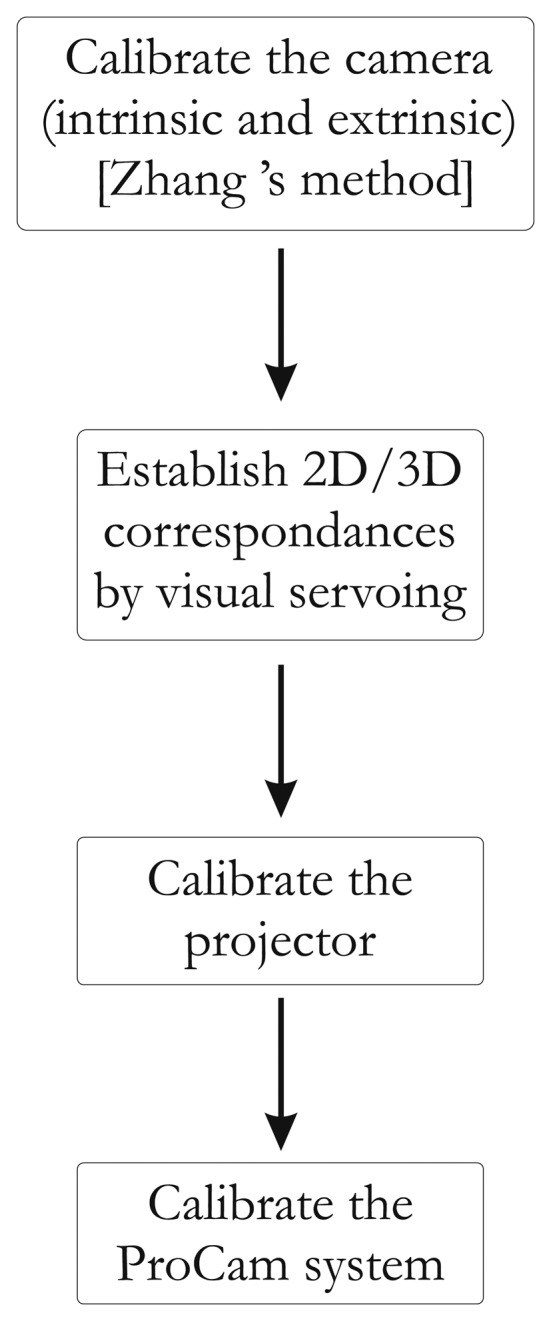
Flowchart summarizing the steps of the full procedure.

Of course, estimating 2D–3D correspondences for the projector is not straightforward. Our method consists in obtaining the 2D–3D correspondences for the projector with a virtual model of the scene coupled with a visual servoing control loop. A global overview of our method is given in Figure 4.

### 2.3. 2D–3D Correspondences for the Projector

**Figure 5.**
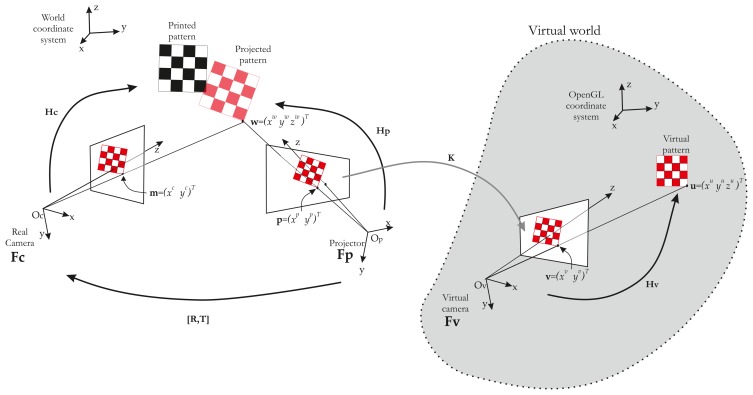
System model: The model consists of three frames representing each device. **F_c_** is the “real” camera frame. This is the camera of the stereo rig-like system. **F_p_** is the projector frame, and **Fv** is the “Virtual” camera or OpenGL camera. The sole transformation between **F_p_** and **F_v_** is their image planes. In other terms, the image plane of **F_v_** is mapped on the image plane of **F_p_** via the graphic adapter.

To recover the correspondences, we use a virtual model (modeled under OpenGL) of our scene. In this virtual world, an “OpenGL camera” takes pictures and sends them to the projector. In other words, the projector shows what the “OpenGL camera” is looking at. If we then use a chessboard pattern, a virtual chessboard pattern is set and the “OpenGL camera” films it. The key idea of our method is to control the “OpenGL Camera” pose by visual servoing, so as to achieve the same rigid motion between *projector/real chessboard* and *OpenGL camera/virtual chessboard*. Thus, we have **H_v_** ∼ **H_p_**. The whole system can be modeled by three frames (Figure 5).

Let us consider a 3D point, **u**, projected in a virtual work space with the coordinates, (*x^u^y^u^z^u^*)*^T^*, expressed by **ũ** in homogeneous coordinates (Figure 5). Let **ṽ** = (*x^v^y^v^*1)*^T^* be the projected point by the virtual camera in the virtual world, such as:

(8) $$\tilde{v}∼H_{v}\tilde{u}∼A_{v}[R_{v}|T_{v}]\tilde{u}$$

where **H_v_** is the 3×4 projection matrix of the OpenGL camera. This is the projection transformation to render the 3D scene (in OpenGL) onto the computer screen as a 2D image. However, the rendering is directly sent to the projector, and so, we have to take into consideration transformation **K** between the image plane of the OpenGL camera and the projector image plane (via the graphic adapter):

(9) $$\tilde{p}=K\tilde{v}$$

In this equation, **K** = **A_p_**.**A_v_**^−1^, where **A_p_** is the projector intrinsic parameter matrix and **A_v_** is the OpenGL camera intrinsic parameter matrix. However, **K** also represents the transformation between the two image spaces. In our case, the OpenGL camera resolution is the same as the projector resolution. Consequently, we can verify that a pixel in the OpenGL camera image plane is the same in the projector image plane and **K** = 
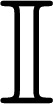
_3_. Thus, Equation (9) can be rewritten as:

(10) $$\tilde{p}=\tilde{v}$$

and:

(11) $$H_{p}\tilde{w}∼H_{v}\tilde{u}$$

From this equation, it is clear that if the vectors, **ũ** and, **w˜** are equal and known, then **H_p_** can be deduced from **H_v_**. **ũ** can easily be extracted from the OpenGL environment, but **w˜** is on the projected pattern. In order to obtain **w˜**, the idea is to superimpose the projected pattern (in red in Figure 5) on a known printed pattern (in black in Figure 5). The fitting of the two patterns is performed by moving the OpenGL camera. The OpenGL is controlled by a visual servoing loop.

The following section describes the fundamentals of visual servoing and breaks down our approach into different steps.

## 3. Visual Servoing Task

### 3.1. Background

In the classical visual servoing approach [18], a camera is mounted on a robot end effector. A visual feedback loop is then closed via the image to control the robot's degree of freedom.

**Figure 6.**
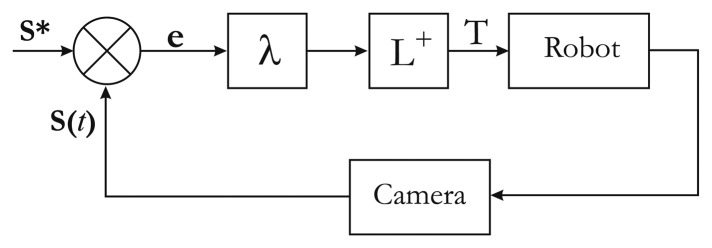
Classical visual servoing scheme.

The aim of classical visual servoing is essentially to minimize error **e**(*t*) between a vector of *n* image sensor features **s**(*t*) seen at the current state and their desired value s* extracted from the current image (Figure 6). This can be expressed as the regulation of function **e**(*t*) called task function and defined as:

(12) e(t)=s(t)−s*

A significant difficulty in minimizing the previous equation is to derive the relation $\dot{s}(t)=\frac{ds(t)}{dt}$, which links the movement of features in the image to the velocity twist of the camera **Θ***_c_*. This relation is defined as:

(13) $$\dot{s}(t)=\text{L}\Theta_{c}$$

where **L** is called the image Jacobian, which represents the mapping from the variation of the camera pose to the variation of a feature in the image.

To minimize error **e**(*t*), an iterative minimization procedure is used. An exponentially decoupled decrease in the error is defined, so that:

(14) $$\frac{de(t)}{dt}=-\lambda e(t)$$

where *λ* is the gain controlling the rate of the decrease of the error. Combining the two previous equations, the velocity twist **Θ***_c_* is obtained, such as:

(15) $$\Theta_{c}=-\lambda L^{T+}e$$

The details of this matrix computation are well documented and are given in [18].

### 3.2. Visual Servoing Applied to Calibration

In our method, the visual servoing task is to fit the printed pattern and the projected pattern. Consequently, the desired value s* is the coordinates of points extracted in the printed pattern and the current value **s**(*t*) is the coordinates of points extracted in the projected pattern. For example, if a chessboard is used, points correspond to corners extracted on the projected pattern.

**Figure 7.**
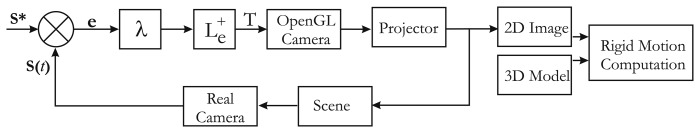
Calibration based on visual servoing.

There is no robot end-effector to control only the OpenGL camera. Moreover, and unlike a classical visual servoing, the OpenGL camera is controlled, but cannot measure the image error **e**(*t*). In contrast, the “real camera” is used to measure **e**(*t*), but is not controlled (Figure 7). Hence, we need to consider the rigid motion between the OpenGL camera (therefore, the projector) and the real camera [**R, t**] (Figure 5). Thus, the image Jacobian must take the rigid motion into account with a new image Jacobian **Le**:

(16) $$Le=L\left[\begin{array}{cc} R & {\left[t\right]}_{\times }R\\ 0_{3} & R \end{array}\right]$$

where [*t*] _×_ is the skew symmetric matrix associated with the translation vector **t**. The image Jacobian **Le***^T^*^+^ is then used in the control law (Equation (15)).

(17) $$\Theta_{c}=-\lambda Le^{T+}e$$

It has been shown that if we consider only a translation between the projector and the real camera, Equation (16) can be rewritten as:

(18) $$Le=L\left[\begin{array}{cc} I & \left[t\right]\\ 0_{3} & I \end{array}\right]$$

Consequently, the convergence $LL_{e}^{+}>0$ is immediately verified. Of course, [**R**, **t**] is unknown, but we have only to ensure that there is no rotation between the camera and projector. In practice, the visual servoing loop is very robust, and it is sufficient to place the camera roughly parallel to the projector. At each iteration, the displacement is computed until the projected pattern fits the printed one.

These steps, which consists in changing the position of the checkerboard, must be repeated (at least 10 times) to obtain different point of views. Finally, we obtain a set of 2D–3D points required to perform the calibration. Matrix **H_p_** is computed by SVD. Equation (5) can also be written as:

(19) $$x^{p}=\frac{H_{p(11)}x^{w}+H_{p(12)}y^{w}+H_{p(13)}z^{w}+H_{p(14)}}{H_{p(31)}x^{w}+H_{p(32)}y^{w}+H_{p(33)}z^{w}+H_{p(34)}}$$

(20) $$y^{p}=\frac{H_{p(21)}x^{w}+H_{p(22)}y^{w}+H_{p(23)}z^{w}+H_{p(24)}}{H_{p(31)}x^{w}+H_{p(32)}y^{w}+H_{p(33)}z^{w}+H_{p(34)}}$$

From Equations (19) and (20), we can write the following linear system (with *n* image to scene point correspondences):

(21) $$FH_{p}=0$$

with:

(22) $$F=\left[\begin{array}{llllllllllll} x_{1}^{w} & y_{1}^{w} & z_{1}^{w} & 1 & 0 & 0 & 0 & 0 & -x_{1}^{v}x_{1}^{w} & -x_{1}^{v}y_{1}^{w} & -x_{1}^{v}z_{1}^{w} & -x_{1}^{v}\\ 0 & 0 & 0 & 0 & x_{1}^{w} & y_{1}^{w} & z_{1}^{w} & 1 & -y_{1}^{v}x_{1}^{w} & -y_{1}^{v}y_{1}^{w} & -y_{1}^{v}z_{1}^{w} & -y_{1}^{v}\\ x_{2}^{w} & y_{2}^{w} & z_{2}^{w} & 1 & 0 & 0 & 0 & 0 & -x_{2}^{v}x_{2}^{w} & -x_{2}^{v}y_{2}^{w} & -x_{2}^{v}z_{2}^{w} & -x_{2}^{v}\\ 0 & 0 & 0 & 0 & x_{2}^{w} & y_{2}^{w} & z_{2}^{w} & 1 & -y_{2}^{v}x_{2}^{w} & -y_{2}^{v}y_{2}^{w} & -y_{2}^{v}z_{2}^{w} & -y_{2}^{v}\\ \vdots & \vdots & \vdots & \vdots & \vdots & \vdots & \vdots & \vdots & \vdots & \vdots & \vdots & \vdots \\ x_{n}^{w} & y_{n}^{w} & z_{n}^{w} & 1 & 0 & 0 & 0 & 0 & -x_{n}^{v}x_{n}^{w} & -x_{n}^{v}y_{n}^{w} & -x_{n}^{v}z_{n}^{w} & -x_{n}^{v}\\ 0 & 0 & 0 & 0 & x_{n}^{w} & y_{n}^{w} & z_{n}^{w} & 1 & -y_{n}^{v}x_{n}^{w} & -y_{n}^{v}y_{n}^{w} & -y_{n}^{v}z_{n}^{w} & -y_{n}^{v} \end{array}\right]$$

and

(23) $$H_{p}=\left[\begin{array}{c} H_{p1}^{T}\\ H_{p2}^{T}\\ H_{p3}^{T} \end{array}\right]$$

where $H_{pi}^{T}$ is the *i^th^* row of H_p_. Thus, **h_p_** can be found by computing the SVD of **F**. Let **F** be **UDV***^T^*. Hence, the last column of **V**, which corresponds to the smallest singular value of **F**, is the solution of Equation (21). Once **H_p_** is known, matrix **A_p_** is found by QR factorization (decomposition of a matrix A into a product A = QR of an orthogonal matrix Q and an upper triangular matrix R). Indeed, **H_p_** = **A_p_** [**R_p_** | **T_p_**], where **A_p_** is an upper triangular matrix (3×3), including the intrinsic parameters, and [**R_p_** | **T_p_**] are the extrinsic parameters.

If we rewrite **H_p_** as:

(24) $$H_{p}=\left[\begin{array}{llll} H_{p11}^{T} & H_{p12}^{T} & H_{p13}^{T} & H_{p14}^{T}\\ H_{p21}^{T} & H_{p22}^{T} & H_{p23}^{T} & H_{p24}^{T}\\ H_{p31}^{T} & H_{p32}^{T} & H_{p33}^{T} & H_{p34}^{T} \end{array}\right]=[\text{M}|H_{p4}]$$

QR factorization applied to **M**^−1^ gives **M**^−1^ = **Q.U**; then **M** = **U**^−1^.**Q**^−1^. **Q** is an orthogonal matrix; then **Q**^−1^ can be identified to **R_p_**. In addition, the inverse of an invertible upper triangular matrix is upper triangular, and so, **U**^−1^ can be interpreted as **A_p_**. Indeed, **U**^−1^ is written as:

(25) $$U^{-1}=\left[\begin{array}{ccc} u_{11} & u_{12} & u_{13}\\ 0 & u_{22} & u_{23}\\ 0 & 0 & u_{33} \end{array}\right]$$

and **A_p_** is like Equation (4). In normalizing by *u*_33_, we obtain:

(26) $$A_{p}=U^{-1}=\left[\begin{array}{ccc} u_{11}/u_{33} & u_{12}/u_{33} & u_{13}/u_{33}\\ 0 & u_{22}/u_{33} & u_{23}/u_{33}\\ 0 & 0 & 1 \end{array}\right]$$

## 4. Experiments

**Figure 8.**
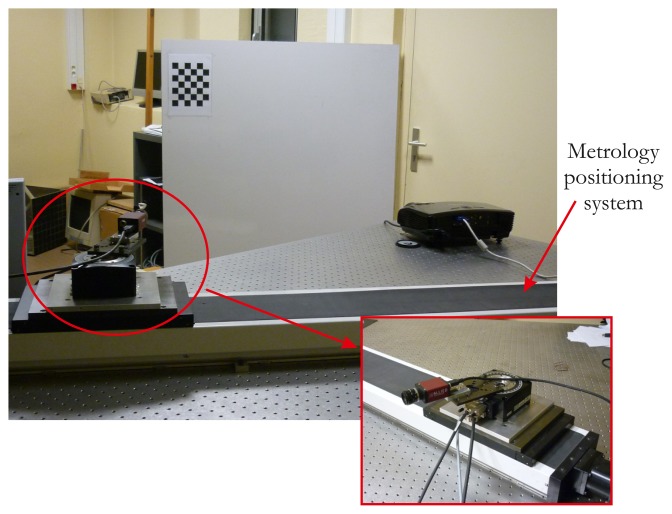
System configuration.

**Figure 9.**
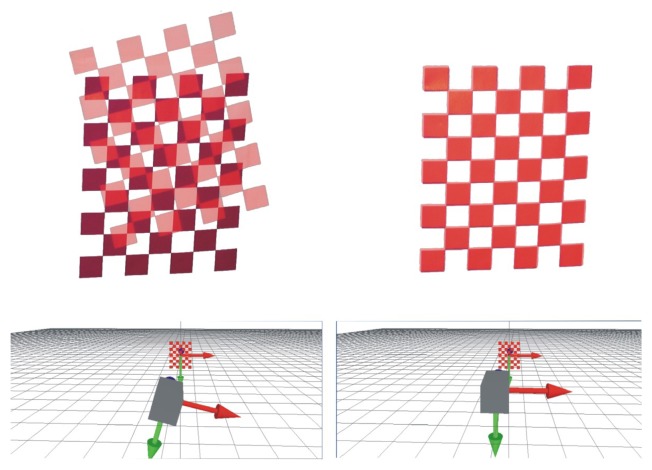
Fitting process example. **Top**: projected view by the virtual camera; **bottom**: virtual workspace.

We assessed our proposed calibration method with a MARLIN F080C camera, which uses the IEEE 1394 protocol (FireWire protocol). Its maximum resolution is 1,024 × 768 pixels. The camera is mounted on a metrology positioning system that allows it to be translated with micrometer accuracy. It consists of a linear and rotating stage from Newport©, controlled by a universal motion driver (Figure 8). The projector is an Optoma VPL-CX75. Its maximum resolution is 1,600 × 1,200 pixels. All software is written in C++ and developed with OpenCV, which provides dedicated tools. The visual servoing task and the creation of the projected pattern are developed in C++ with the OpenGL library (Figure 9). The camera image acquisition is also coded in C++, and the whole system is run on Linux Kubuntu©.

### 4.1. Procedure

We will compare our method with the two other most commonly used techniques, that of Audet and Okutomi [15], and the plane-based methods [3–6]. The latter also use a checkerboard. Our method is organized as follows:

1. Set up the position of the projector and camera.
2. Calibrate using the three different methods.
3. Translate the camera (10 cm) with the calibration bench.
4. Recalibrate the system with each method.
5. Measure the translation error with the rigid motion matrix.

The first step is considered crucial. Many calibration methods for camera–projector systems need a special set-up. For instance, the camera must be carefully set and placed, since it can acquire frames containing the projected and printed patterns for the plane-based method. Thus, it must have a large field of view or be correctly placed to acquire all the frames needed. In another approach [15], the camera and projector must be properly positioned, otherwise calibration cannot be performed. In methods using structured light patterns for establishing correspondences, the set-up is less important than with other methods, but the camera still needs to be focused on the printed pattern for accuracy. In our method, no special set-up is required; the field of view can be wider than the scene projection without accuracy being greatly affected. In view of these limitations, it is not possible to have a single location for each calibration method. With each technique, it takes time to properly set up all the devices so that the performance of each algorithm is maximized. The translation error is measured along the x-axis, such as:

(27) $$\text{Error}=T_{x}-T_{x+10cm}$$

Four parameters were used to assess each method: intrinsic parameter characterization, translation error, numbers of frames needed and ease of use.

### 4.2. Results

A typical example of the visual servoing process implemented in our method is given in Figure 8. We can observe in this image the starting position of the projected pattern and, then, the result when it is totally fitted on the printed one. Calibration is performed by repeating this process at least 10 times with different orientations of the printed pattern. The results of each method assessed are given in Table 1.

**Figure 10.**
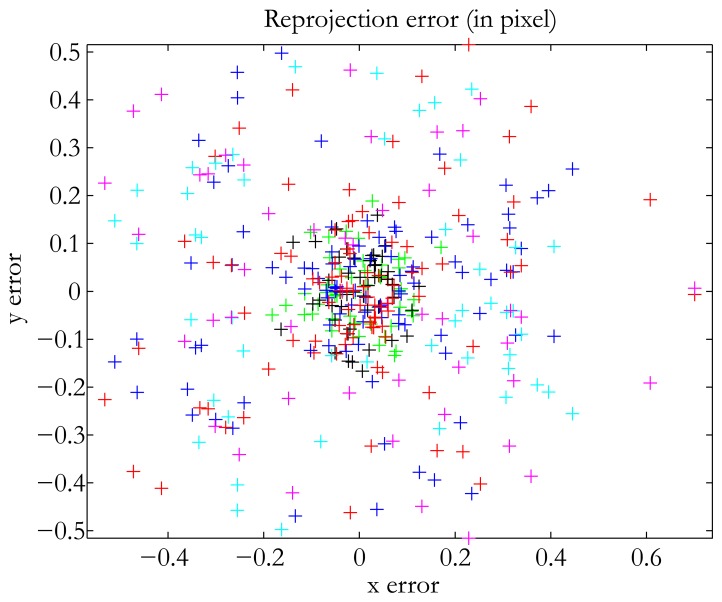
Reprojection error: Difference in pixel coordinates between the position of grid corners observed in the image and that predicted by the computed calibration parameters. Each color corresponds with the position of a checkerboard. In this figure, only six positions have been considered for the purpose of clarity.

In terms of accuracy, our method falls in between the two already existing methods. The translation error was 0.47 mm, which is good when dealing with structured lighting applications, for instance. We used the same number of images as Audet and Okutomi. Of course, the addition of more images increases accuracy. The best result we obtained was 0.3 mm with 17 images. Our method was the easiest to use, since it does not impose any constraints on the position of the devices. In addition, we used no additional material, and the calibration pattern was a classical checkerboard. The method of Audet and Okutomi [15] was the most accurate of the three in calibrating the projector-camera system. The translation error was very low, as was the reprojection error. However, as indicated by the authors themselves, their method has one major drawback: the calibration board must be placed at angles of approximately 45° in relation to the image planes of the projector and the camera to maximize accuracy. Thus, the user must have experience in calibration tasks to perform the method satisfactorily. The plane-based method was the least accurate of the three tested. It is also the least user-friendly, because it takes a long time to process all the images. Finally, a quick comparison with the results obtained by the authors cited in the introduction shows that our method is not badly placed in terms of reprojection error for the projector (see Table 1). Figure 10 shows the distribution of our reprojection error.

## 5. Conclusions

We present a new calibration method for projector-camera systems based on visual servoing. This technique simplifies the process, since the same 3D points are used for both camera and projector calibration. The method is simple and requires no special hardware or calibration artifacts. The experimental results show that the accuracy of the method is comparable to that of existing techniques. In the future, we would like to develop an adaptive gain for the visual servoing task to improve time processing at the end of the fitting process. Another feature we would like to investigate is the effect of the initial pose of the projected pattern. At present, this position must be set manually, and so, if the projected pattern is far from the printed pattern, the process will take longer.

## Figures and Tables

**Table 1.** Calibration results.

**Method Tested**	**Audet *et al.* [15]**	**Plane-Based**	**Our Method**
**Focal length**	1,956	1,984	1,948
**Reprojection error (projector)**	0.25	1.18	0.38
**Translation error**	0.25 mm	8.9 mm	0.47 mm
**Number of frames**	10	20	10
**Useability**	++	+	+++
